# Evaluation of different DNA extraction methods based on steel‐bullet beating for molecular diagnosis of onychomycosis

**DOI:** 10.1002/jcla.24657

**Published:** 2022-08-21

**Authors:** Marjan Motamedi, Abdulbaqi Amini, Somayeh Yazdanpanah, Mozhgan Mahmoodi, Hossein Khodadadi, Hamidreza Zalpoor

**Affiliations:** ^1^ Department of Parasitology and Mycology, School of Medicine Shiraz University of Medical|Science Shiraz Iran; ^2^ Neuroscience Research Center Shiraz University of Medical Siences Shiraz Iran

**Keywords:** bullet‐beating, DNA extraction, ex vivo model, onychomycosis, PCR

## Abstract

**Background:**

Considering increased trends toward molecular methods for detection/identification of fungi causing onychomycosis, the aim of this study is comparison three DNA extraction methods based on steel‐bullet beating to extract DNA from nail.

**Methods:**

Ex ‐vivo onychomycosis model was developed using bovine hoof with *Candida albicans* and *Aspergillus flavus*. For two models, total DNA was extracted using the three different methods. In method 1, the extraction and purification were performed by steel‐bullet beating and phenol chloroform protocol, respectively. In method 2, a freezing step were applied before beating. The purification step in method 3 was carried out using a commercial kit, although DNA extraction was done similarly to method 1 in that approach. To evaluate the efficacy of each method, the extracted genomic DNA was amplified with Polymerase Chain Reaction (PCR) using Internal Transcribed Spacer (ITS) regions. Moreover, 50 nail samples were evaluated for onychomycosis using direct microscopy examination as well as PCR in order to evaluate the diagnostic efficiency of the optimal DNA extraction method.

**Results:**

Regarding the desirable quality of the extracted DNA, cost effectiveness, and simplicity, method 1 could be used to extract DNA effectively. Additionally, the obtained data showed that PCR had a higher detection rate of fungal agents in the nail samples than direct microscopic examination.

**Conclusions:**

This study demonstrated that the mechanical disruption of the cell wall by steel‐bullet beating is a useful and practical method to improve the quantity and quality of fungal DNA thorough the extraction process.

## Graphical abstract

DNA extraction from nail fragments to diagnose onychomycosis based on PCR‐based methods. Extraction of the fungal genomic DNA from nail fragments suspected to onychomycosis using steel‐bullet beating is an efficient and applicable method to PCR analysis in order to detecting and screening the fungal pathogens that cause onychomycosis.

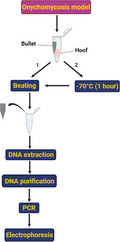

## 1 INTRODUCTION

Fingernails and toenails not only serve as protection for the surrounding soft tissues with their sensory and mechanical functions but also show a visual perspective of a person's overall health. Onychomycosis is a fungal nail infection that may involve any parts of the nail unit and is responsible for about 50% of all consultations for nail disorders.^1^ Onychomycosis more frequently affects toenails compared with fingernails and is characterized by nail thickening, splitting, roughening, and discoloration.^2^ In most cases, this infection is caused by anthropophilic dermatophytes (60%–70%), in particular *Trichophyton rubrum* and *Trichophyton interdigitale*. Such yeasts as *Candida albicans* and *Candida parapsilosis*, and nondermatophyte molds like *Aspergillus* spp. are including the other causative agents of onychomycosis.^3^, ^4^ In most cases, long‐term antifungal therapy is needed due to chronicity and recurrence of onychomycosis.^5^ Consequently, an accurate species identification of fungi that is responsible for infection is essential for selecting an appropriate treatment because of a diversity of causative agents and different susceptibility to antifungal drugs.^6^

Microscopic examination and fungal culture are the gold standard methods for diagnosis of onychomycosis, but high false‐negative results have resulted in a turn techniques with more sensitivity and specificity such as polymerase chain reaction (PCR).^7^ Compared with culture‐based techniques, PCR‐based methods could able to detect fungal genomic DNA in infected nail tissue even with low fungal load. So, the application of PCR methods for detection of fungal agents can lead to the prompt diagnosis of onychomycosis with more sensitivity.^8^ All in all, the PCR technology had a major role in many aspects of onychomycosis including diagnosis,^9^ identification of etiological agents,^10^ and epidemiology.^11^

One of the principal steps in the performance of PCR‐ based diagnostic assays is DNA extraction from the targeted infectious agents with high quality and quantity that leads to the successful diagnosis of infectious diseases.^12^ Up to now, a variety of methods with different approaches have been established to isolate DNA from biological specimens.^13^ Considering the hard structure and keratinized tissue of nails, and also the lack of a standardized method for DNA extraction from the nail samples, most molecular studies on fungal nail infections have made use of commercial kits to achieve this intention.^14^, ^15^, ^16^ So, evaluation of different extraction methods using relatively common reagents and devices in a laboratory could be helpful for the diagnosis of fungal nail infections. In view of these considerations, a comparative assessment was conducted on three methods based on steel‐bullet beating to achieve an efficient, sensitive, rapid, and simple method for extracting fungal genomic DNA from nail fragments.

## 2 MATERIALS AND METHODS

### 2.1 Ex vivo onychomycosis models

In order to survey different methods of DNA extraction based on steel‐bullet beating, we needed a huge, distinctive, and homogeneous sample. To obtain the sample, and also with regards to the previous studies, hooves were applied as a suitable sample for implementation of the onychomycosis model in this study.^17^, ^18^, ^19^

Since both yeasts and molds agents are involved in onychomycosis, two models were prepared in this survey including the onychomycosis model infected by *Candida albicans* ATCC 5982 (model 1) and the other by *Aspergillus flavus* ATCC 64025 (model 2). For this purpose, bovine hooves from freshly slaughtered bovine, free of adhering connective and cartilaginous tissues, were soaked in phosphate‐buffered saline (Sigma) for 24 h. Then, slices of thickness of about 450–600 μm were cut from the distal part of the hoof using a microtome (Leitz 1512). The hoof slices were sterilized by autoclave method at 121°C for 30 min and were placed on a previously sterilized microscope slide. An inoculum of the fresh colonies grown on Sabouraud Dextrose Agar (SDA) (Difco) was inoculated onto the sterilized hoof fragments. Another piece of hoof slice was placed on top of the previous piece, so that the inoculated fungal colonies were sandwiched between the two pieces of hoof slices. The slide was placed on a U‐shaped glass tube in a sterile petri dish containing distilled water. Finally, the Petri dish was kept at 25°C for 2 weeks and at 37°C for 1 week to create the onychomycosis model with *A. flavus* and *C. albicans*, respectively. After incubation, in order to confirm the implementation of the onychomycosis model, the contaminated hoof was scraped and stained with calcofluor white (CFW) (Thermo Fisher) to evaluate via a fluorescent microscope (Olympus BX61).

### 2.2 DNA extraction

Genomic fungal DNA was extracted according to three methods as follows:

Method 1: At first, a sterile conical steel bullet was inserted into a 2 ml Eppendorf tube containing a fragment of hoof sample (approximately 20 mg). Then, the tube was beaten with repeated blows for 5 min until converting the sample to powder. After that, the bullet was washed with 200 μl lysis buffer (100 mM NaCl, 1 mM EDTA, 10 mM Tris–HCl, 2% Triton X100, and 0.5% SDS) and put out from the tube. The next step included DNA purification using the conventional phenol‐chloroform protocol.^20^ In brief, 200 μl phenol‐chloroform was added to the tube containing the sample, lysis buffer, and extracted DNA. The mixture was then centrifuged at 5000 rpm for 5 min. In the following, the supernatant that contains extracted DNA was transferred to a new sterile tube. To continue, isopropanol in the same volume as the supernatant and sodium acetate (pH 5.2) in one‐tenth of the volume of the supernatant were added to the supernatant. After incubation at −20°C for 1 h, the mixture was centrifuged at 12,000 rpm for 15 min. The precipitant was transferred to another tube and washed with cold 70% ethanol and dried in air. Finally, the dried precipitant, containing genomic DNA, was mixed with 50 μl of pyrogen‐free water and stored at −20°C until use.

Method 2: DNA extraction and purification steps were performed as described above in method 1, with a difference that the tube containing sample and bullet was frozen at −80°C for at least 1 hour before being beaten.

Method 3: DNA extraction step was done similar to method 1, but the purification step was performed using a commercial kit (Yekta Tajhiz Azma, Iran) according to the manufacturer's protocol.

Control method: In addition to the three above‐mentioned extraction methods using bullets, beating with glass beads was used as a control method that has been used in molecular studies on fungi, extensively.^21^ To do so, nearly 300 μl of 0.5 mm diameter acid‐washed glass beads (Sigma) was added to each Eppendorf tube containing the sample and 200 μl lysis buffer, and the tube was heavily shaken for 3 min. Following this, the DNA purification step was performed in accordance with the phenol‐chloroform protocol.

### 2.3 Assessment of extracted DNA

Quantification of extracted DNA was determined through the nanodrop‐spectrophotometer (Thermo Scientific). So, the purity (absorbance ratio at 260/280 nm) and concentration (μg/ml) of the extracted DNA were measured.

### 2.4 PCR amplification

Amplification of internal transcribed spacer (ITS) of rDNA region in fungal species was applied indicatively in order to prove the existence of fungal DNA in the onychomycosis models.

The ITS region of each extracted DNA was amplified in a Thermal Cycler (Bio‐Rad) using ITS1 (5‐TCCGTAGGTGAACCTGCG‐3) and ITS4 (5‐TCCTCCGCTTATTGATATGC‐3) primers. The reactions consisted of 0.25 μl (50 pmol) of each primer, 12.5 μl of PCR master mix (Ampliqon RED), 5 μl of extracted DNA, and 7 μl of sterile distilled water in a final reaction volume of 25 μl. PCR program was performed as follows: initial denaturation at 95°C for 6 min; 35 cycles of denaturation at 94°C for 45 s, annealing at 58°C for 1 min, and extension at 72°C for 1 min; using PCR system 9600 thermal cycler (BIORAD). Extracted DNA of both onychomycosis models by all applied protocols were evaluated by PCR. The PCR products were analyzed by agarose gel electrophoresis using 1% agarose gel (Thermo Fisher). Electrophoresis was then performed using 1 × Tris–Borate EDTA (TBE) buffer containing 1 μg/ml of ethidium bromide (EtBr) and a constant voltage of 100 V for 50 min. The DNA bands were visualized using a UV detector (UVITEC).

### 2.5 Cost and time estimation

The cost of each extraction method was estimated by summing up the costs of the chemical reagents, commercial kits, and disposable/reusable laboratory instruments used. The minimum time required to complete DNA extraction for each method was estimated from the beginning of the procedure to its end.

### 2.6 Clinical specimens

Fifty nail samples were collected from the infected nails of the patients clinically suspected of onychomycosis. Each sample was split into two parts; one for direct microscopy examination by potassium hydroxide, and the other one for PCR following the optimal DNA extraction method was determined in this investigation.

### 2.7 Statistical analysis

Statistical analysis of data was performed by the Kruskal–Wallis test for comparing multiple groups and by the Mann–Whitney U test for comparing paired groups. The results were expressed as mean ± Standard Error of Mean (SEM). All statistical analyses were performed using the SPSS statistical software (version 10.0, SPSS Inc, IBM). *p* values ≤0.05 was considered statistically significant.

## 3 RESULTS

CFW staining of both onychomycosis models revealed the establishment of fungal infection by *C. albicans* and *A. flavus* in hoof samples. The concentration and purity of the extracted DNA resulted in each of the methods have been summarized in Table 1. According to our results, purity and concentration of extracted DNA using different extraction methods had no significant difference, statistically. However, the highest and lowest concentrations of DNA obtained from both models that were measured by nanodrop‐spectrophotometer were observed in method 2 (591 ± 291) and control method (171 ± 84), respectively. Moreover, the highest purity was obtained using method 3 for the infected model by *A. flavus*. Also, the concentration of DNA extracted from the onychomycosis model infected by *A. flavus* was significantly higher than *C. albicans* (*p* value ˂0.001). Nonetheless, DNA with no significant difference in purity was obtained from both created onychomycosis models (*p* value: 0.16).

**TABLE 1:** Comparison of the averaged values of DNA purity and concentration yielded by different DNA extraction methods and estimated execution time for each method

Method		Onychomycosis models	DNA purity (Mean ± SE)	DNA concentration (Mean ± SE) μg/ml	Time estimation
Extraction	Purification				
Method 1					
Steel bullet + lysis buffer	Phenol chloroform	Model 1	1.91 ± 0.10	244 ± 31.27	≃80 min
		Model 2	1.84 ± 0.52	490 ± 54.82	
Method 2					
Freezing + steel bullet+ lysis buffer	Phenol chloroform	Model 1	1.74 ± 0.01	366 ± 49.69	≃140 min
		Model 2	1.93 ± 0.04	884 ± 83.56	
Method 3					
Steel bullet	Commercial kit	Model 1	1.77 ± 0.09	169.2 ± 27.94	≃65 min
		Model 2	1.3 ± 0.18	440 ± 50.82	
Control method					
Glass beads + lysis buffer	Phenol chloroform	Model 1	1.86 ± 0.12	117 ± 32.48	≃80 min
		Model 2	1.83 ± 0.05	262 ± 39.75	

**FIGURE 1:**
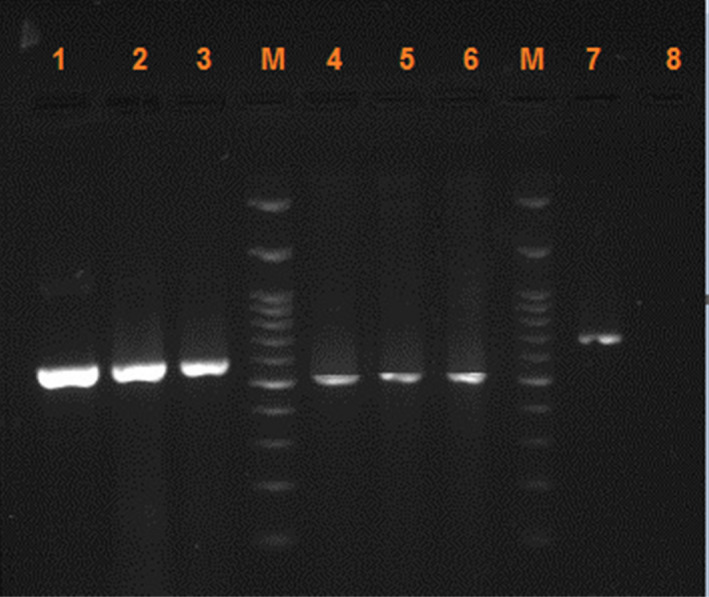
Examples of the electrophoretic patterns of PCR product by ITS primers. Lanes 1, 2, 3: *Aspergillus flavus* (model 2) using methods 1, 2, and 3, respectively. Lanes 4, 5, 6: *Candida albicans* (model 1) using methods 1, 2, and 3, respectively. Lane 7: *Trichophyton rubrum* as the positive control. Lane 8: negative control, and Lane M: 100 bp. DNA marker

The quality of the PCR products by agarose gel electrophoresis have shown in Figure 1. As the figure depicts, amplification the extracted DNA using the primer set ITS1‐ITS4 resulted in products varied in size from 550 to 630 bp. The target DNA extracted by beating with a steel bullet was successfully amplified in both onychomycosis models. Additionally, the ITS PCR amplicons generate no smears on the agarose gel for any of the products.

In this study, the total time of the extraction for each method was estimated. The freezing and beating methods in the extraction step took 60 and 5 min, respectively. The phenol‐chloroform and commercial kit methods in the purification step took 75 and 60 min, respectively. Therefore, the maximum and minimum times for complete procedure were related to method 2 (140 min) and method 3 (65 min), respectively (Table 1).

Regarding our findings in the current study, the DNA extraction method based on beating by steel bullet was more cost‐effective compared with beating by glass beads, because the steel bullets are reusable after washing with alcohol and sterilizing with an autoclave. Furthermore, method 1 has no need to freeze the sample, and purification was done by common reagents. So, this method is simple with the capability for performance in most laboratories with fewer costs compared with other studied methods.

Comparison of the proportion of true‐positive results in clinical specimens of onychomycosis diagnosed by direct microscopic examination and PCR demonstrated that PCR resulted in the detection of 72% of cases (36/50), whereas 52% of the samples (26/50) was found positive in microscopic examination.

Importantly, PCR led to the detection of fungal agents in 12 cases of onychomycosis in which their microscopic examination was negative (Table 2).

**TABLE 2:** Results of direct microscopic examination and PCR for 50 clinical samples of onychomycosis

	Number (percentage)		Direct microscopic	PCR
Relationship between tests				
Match tests	36 (72%)	24 (48%)	Positive	Positive
		12 (24%)	Negative	Negative
Mismatch tests	14 (28%)	12 (24%)	Negative	Positive
		2 (4%)	Positive	Negative
Total sample	50 (100%)		—	—

## 4 DISCUSSION

An accurate diagnosis of onychomycosis is an important prerequisite for proper and successful treatment. Molecular biology tools are increasingly being used to overcome the poor diagnostic sensitivities and long turnaround times associated with the detection and identification of fungal pathogens in clinical samples such as nails.^7^ Application of different methods of sample storage and collection, DNA extraction, sequencing library preparation, and bioinformatics analysis has been shown to contribute variability to the results of molecular studies. In this regard, the extraction of high‐quality nucleic acids is among the most important processes, since it can introduce bias at the initial step.^22^ According to the study by Pankewitz et al., the optimization of DNA extraction from nail specimens is one of the key parameters to obtain higher sensitivity in molecular assays.^23^

Up to now, few studies have investigated the DNA extraction process from nail samples,^22^, ^24^, ^25^ while multiple studies have focused on the diagnosis of fungal nail infection based on PCR methods by detecting the fungal DNA from nail samples, directly.^15^, ^26^, ^27^ Due to the tough and firm nature of the specific nail structure, it seems to be helpful to introduce an efficient, sensitive, rapid, simple to use, and cost‐effective protocol for the extraction of nucleic acids from it. Another crucial point for DNA extraction from fungal agents is the presence of a strong structure of cell walls that enhance their toughness. These structures usually require a combination of freezing and beating, and strong buffers for the cell walls to be broken for the DNA to be successfully extracted.

The published literature has suggested that the complete lysis of fungal cell walls through beating can make a significant impact on yielded results.^28^ Beating is a mechanical method to disrupt the cell wall that is performed prior to standard DNA extraction. In this step, ceramic or glass beads are added to the tube containing clinical samples. This is followed by moderate to high‐speed shaking, causing heavy collisions between the beads and the samples. A number of different beating protocols have been used to extract fungal DNA and RNA from clinical samples suspected of fungal infections.^21^, ^29^ The current study compared three extraction methods based on steel‐bullet beating in the DNA extraction step to identify how to produce the highest yield of ribosomal DNA for PCR. Conical steel bullet was used for the first time as a tool for extracting DNA from the clinical samples taken from the patients suspected of dermatophytosis to survey the diagnostic performance of a pan‐dermatophyte real‐time PCR assay.^30^

The results of this study demonstrated that the mechanical disruption of the cell wall by steel‐bullet beating was a successful method to improve the quantity and quality of fungal DNA during the extraction process from yeasts and molds. The only advantage of the control method using glass beads compared with the other three methods was the lack of need for reusing glass beads, which was accompanied by a low risk for accidental contamination. It should be noted that the possible flaw of contamination in the use of steel bullets could be managed by washing the steel bullet with alcohol and autoclaving it after each usage.

The differences in the amounts of fungal DNA recovered with different extraction methods detected by PCR in the present study highlighted the importance of the extraction step. What follows includes the comparison of the three methods. The difference between method 1 and method 2 was in the extraction stage. In method 2, incubating the sample at −80°C for 1 h before extraction was added to the steps of the process. It is noteworthy that freezing the samples at −80°C was used instead of liquid nitrogen which has such disadvantages as being expensive to purchase, being maintained in proper conditions, and having hazards for use. As expected, the addition of this step made the fungal cell wall more vulnerable to lysis. As a result, a higher concentration of DNA was extracted that is in contrast to the findings of the research by Scharf et al., which indicated that the exposure of specimens to liquid nitrogen did not lead to more effective lysis of the fungal cells.^21^ Nevertheless, method 1 was chosen due to being more practical considering the time‐consuming freezing step in method 2.

The main difference between the steps of method 1 and method 3 was in the purification step, for which phenol‐chloroform and commercial kit were used, respectively. Although the application of commercial kits is quick and easy, especially when working with a large number of samples, no significant difference was observed in the DNA purification rate. So, method 1 was considered more advantageous.

Among the three methods based on steel‐bullet beating, most differences in the work procedures were observed between methods 2 and 3, because there were differences in both DNA extraction and purification stages. Although using method 3 saved more time compared with method 2, the latter was introduced as the superior method because of the higher concentration of the extracted DNA and the lower cost of consumption.

Due to the different cell wall structures of yeasts and molds, the efficiency of DNA extraction is highly variable.^31^ An ideal extraction method should accurately recover DNA from a wide variety of fungi and avoid the bias that can be introduced by incomplete cell wall disruption. Although a higher DNA yield was obtained in model 2 (*A. fumigatus*) than in model 1 (*C. albicans*) in the current study, both yeast and molds could be detected in onychomycosis models submitted for PCR diagnostic assays. Differences between the concentration of extracted DNA from yeast and mold have also been reported in other previous studies.^21^, ^31^

Overall, some advantages and disadvantages must be acknowledged for all studied methods. However, method 1 was the most suitable for direct DNA extraction from nail samples regarding different aspects such as the concentration and quality of the extracted DNA, PCR band quality, time consumption, cost‐effectiveness, labor use, and simplicity.

Using clinical isolates instead of reference strains has been encouraged by former findings, since reference strains have lost their pathophysiological characteristics during long‐term cultivation^32^ that may have an impact on the DNA extraction efficiency. In order to evaluate the diagnostic efficiency of the considered DNA extraction method (method 1) in the present study, the direct microscopic results of 50 nail samples were compared with their PCR results. The indicative application of the pan‐fungal ITS PCR in the nail samples showed that fungal DNA was successfully extracted by the intended method. Moreover, obtained results confirmed an increased detection rate of fungal agents in the nail samples by PCR compared with direct microscopic examination. In this regard, the concordance between direct microscopy and PCR was 72%.

In the current research, a reliable, quick, and inexpensive ex vivo onychomycosis model was implemented using bovine hoof slices to achieve a homogeneous sample for investigating different DNA extraction methods. The first report of the bovine hoof model for onychomycosis was described by Monti et al. in 2011 for the evaluation of topical antifungal activity.^19^ The model employed in the present study encouraged fungal agents to invade the deeper layers of the bovine hoof which was confirmed by a fluorescent microscopic examination. However, the drawback of this study was not using dermatophyte fungi in the implemented onychomycosis ex vivo model which have been classified as the leading causative agent of onychomycosis in some studies.^33^, ^34^

In conclusion, steel‐bullet beating could be effectively employed to perform downstream PCR analysis for detecting and screening the fungal pathogens that cause onychomycosis regarding the appropriate quantity and quality of the extracted DNA using a simple and cost‐effective method with saving time and labor. Finally, it is recommended to evaluate the efficiency of this method to extract DNA from other infected tissues or other microorganisms.

## AUTHOR CONTRIBUTIONS

Marjan Motamedi conceptualized the study, analyzed, and interpreted the data; Abdulbaqi Amini, Somayeh Yazdanpanah, and Mozhgan Mahmoodi performed the study and acquired the data; Somayeh Yazdanpanah and Hamidreza Zalpoor prepared and edited the article; Marjan Motamedi and Hossein Khodadadi reviewed the article and supervised the study.

## FUNDING INFORMATION

This work was supported by the Deputy of Research and Technology of Shiraz University of Medical Sciences, Shiraz, Iran (grant No. 17206).

## CONFLICT OF INTEREST

None of the authors have any relevant conflicts of interest in the studies and data in this article.

## CONSENT FOR PUBLICATION

Informed consent of all authors.

## Data Availability

All data and materials are available.
